# Motor Unit Discharge Patterns in Response to Focal Tendon Vibration of the Lower Limb in Cats and Humans

**DOI:** 10.3389/fnint.2022.836757

**Published:** 2022-04-26

**Authors:** Christopher K. Thompson, Michael D. Johnson, Francesco Negro, Dario Farina, C. J. Heckman

**Affiliations:** ^1^Department of Health and Rehabilitation Sciences, Temple University, Philadelphia, PA, United States; ^2^Department of Physiology, Northwestern University, Chicago, IL, United States; ^3^Department of Clinical and Experimental Sciences, Università degli Studi di Brescia, Brescia, Italy; ^4^Department of Bioengineering, Imperial College London, London, United Kingdom

**Keywords:** motor unit, tendon vibration, electromyogram (EMG), reflex, cat, human

## Abstract

High-frequency vibration of the tendon provides potent activation of Ia afferents time-locked to the stimulation frequency and provides excitatory ionotropic activation of homonymous motor pools. In cats, the evoked motor unit discharge is constrained to discharge at integer multiples of the vibration frequency, resulting in a probability of discharge that is highly punctuated. Here we quantify the robustness of this punctuated response in the cat and evaluate whether it is present in the human. Soleus electromyography (EMG) was collected from eight cats using 64 channel electrodes during three modes of motoneuron activation. First, tendon vibration parameters were modified. Second, secondary reflex inputs are applied concurrently with tendon vibration. Third, the state of the spinal cord was altered through pharmacological or surgical manipulations. Analogous surface high-density EMG was collected from the lower leg of six humans during both vibration evoked and matched volitional contractions. Array EMG signals from both the cat and human were decomposed into corresponding motor unit action potential spike trains, and the punctuation in discharge was quantified. In the cat, regardless of vibration parameters, secondary synaptic drive, and state of spinal circuitry, focal tendon vibration evoked punctuated motor unit discharge. However, in the human lower limb, the vibration-evoked contractions do not produce punctuated motor unit discharge.

## Introduction

Focal vibration of the tendon is used to activate the homonymous motor pool in both animals and humans, and it results in potent activation of the muscle spindles (Brown et al., 1967). Spindles respond to vibration with a stereotypical Ia discharge, which is one-to-one with the period of the vibration frequency. For example, in the cat, vibration up to 500 Hz results in Ia afferents to discharging time-locked to the vibration frequency (Granit and Henatsch, 1956; Brown et al., 1967). In humans, Ia afferent discharge may be locked to either the vibration frequency or its subharmonics (Burke et al., 1976; Houk, 1976). These afferents activate the motoneuron pool in a one-to-all pattern, where a single Ia afferent provides excitatory monosynaptic input to nearly every motoneuron in the pool (Mendell and Henneman, 1968). At the level of the motoneuron, this induces membrane depolarization at the vibration frequency (Westbury, 1972) and can activate persistent inward currents (PIC; Lee and Heckman, 1996). As such, tendon vibration can evoke a tendon vibration reflex (TVR), where the vibration of the distal tendon can evoke motor output through activation of monosynaptic Ia afferents.

Recordings from the afferents, the motoneuron, and the muscle are routinely made during these vibration evoked contractions, however, the resulting discharge patterns of individual motoneurons or motor units are less commonly reported. In the cat, there is evidence that tendon vibration produces motor output by inducing highly synchronous discharge at integer multiples of the vibration frequency, resulting in a probability of discharge that is highly punctuated (Matthews, 1966; Homma et al., 1971; Thompson et al., 2018). Though this result is strikingly apparent in the cat in response to tendon vibration, it remains unclear how or if this rule-like motor unit response to tendon vibration can be modified.

Observations in humans are less uniform. In fact, frequency-locked discharges have been observed occasionally in the arm (Romaiguere et al., 1991), leg (Homma et al., 1971; Burke and Schiller, 1976), and jaw muscles (Desmedt and Godaux, 1975; Hagbarth et al., 1976) of humans. Nevertheless, the majority of human investigations of motor unit activity during TVR of limb muscles do not observe (Godaux et al., 1975; Hagbarth et al., 1976) or report (Bongiovanni et al., 1990; Bongiovanni and Hagbarth, 1990; Romaiguere et al., 1993; Kiehn and Eken, 1997; Griffin et al., 2001; Gorassini et al., 2002, 2004; Grande and Cafarelli, 2003; MacDonell et al., 2010; Fuglevand et al., 2015; Mosier et al., 2017) rigid time-locked motor unit discharge.

Motor unit discharge patterns provide one of the most detailed measures of the mammalian motor system. Given our initial observations of punctuated discharge in the cat (Thompson et al., 2018), we first sought to test how rigid this punctuated discharge is in the cat. We did so by varying the vibration parameters, adding secondary reflex drive, and altering the state of spinal circuitry. In the cat, in all cases and in all units, punctuated discharge in response to tendon vibration was observed. We then sought to quantify motor unit discharge in response to tendon vibration in the human using high density EMG approaches. In the human, TVR evoked motor unit discharge is not punctuated.

## Materials and Methods

### Cat

#### Ethical Approval

Data presented here are from eight adult cats of either sex weighing 2.5–4 kg. All animals were obtained from a designated scientific research breeding establishment. Animals were housed at Northwestern University’s Center for Comparative Medicine, an AAALAC accredited animal research program. All procedures were approved by the Institutional Animal Care and Use Committee at Northwestern University.

#### Terminal Surgery

Anesthesia was induced with 4% isoflurane and a 1:3 mixture of N_2_O and O_2_. A permanent tracheal tube was placed through which isoflurane (0.5–2.5%) and gasses were delivered for the duration of the procedures. The animal was immobilized using a stereotaxic frame by a head clamp, a spinal clamp on the L2 dorsal vertebral process, and bilateral hip pins at the iliac crest. The left hindlimb was secured through pins at the knee and clamps at the ankle, and the right hindlimb was secured using a clamp distal to the ankle. The left soleus was dissected, isolated, and its distal tendon was attached to a load cell via a calcaneus bone chip in series with a linear variable differential transformer and customized voice coil. A distal, cutaneous branch of the right superficial peroneal nerve was dissected, and a cuff electrode was secured around the nerve. Likewise, in one experiment, the left common peroneal nerve was dissected and secured with a cuff electrode proximal to the innervation of the left TA. On select experiments, a L4-S1 laminectomy was provided for intrathecal drug administration via subdural catheter. The dorsal and ventral roots were left intact. In all experiments, following a craniotomy, a precollicular decerebration was performed. At this point, the animals are considered to have a complete lack of sentience, and anesthesia was discontinued (Silverman et al., 2005). An esophageal thermistor assisted in the maintenance of heat lamps and hot pads to maintain a core temperature of 35–37^°^C. At the end of the experiment animals were euthanized using a 2 mM/kg solution of KCl in addition to a bilateral thoracotomy.

#### Electromyography and Force Recordings

Differential EMG recordings were collected using a custom 64-channel array electrode (5 × 13; 2.54 mm interelectrode distance) placed on the surface of the exposed soleus muscle. A ground electrode was placed on the back. Array data were filtered (100–900 Hz), amplified (0.5–2k), and sampled at 5,120 Hz by a 12-bit A/D converter simultaneously with soleus force data (EMG-USB 2, OT Bioelettronica, Torino, Italy).

#### Sensory Inputs

Vibration was delivered at high frequencies (∼130 Hz) and small amplitude (∼80 μm) through the voice coil. This device was fit with a linear variable differential transformer to directly measure linear position of the voice coil. In one experiment, a child size electric toothbrush (Colgate) was manually applied to the distal tendon of the soleus. Such manual application that is perpendicular to the tendon is similar to the approach used in humans.

The role of additional synaptic inputs on the punctuated discharge of motor units during tendon vibration was assessed using three forms of inputs in three separate animals. First, muscle stretch was delivered during tendon vibration through the same voice coil using a low frequency (1 Hz), and large amplitude (1 cm) imposed sin wave change in muscle length. Second, crossed extension reflex was evoked through stimulation (20–50 Hz; 1-ms biphasic; 2× reflex threshold) delivered to the contralateral superficial peroneal nerve through the cuff electrode using a Grass S88 stimulator and isolation unit. Third, reciprocal inhibition was evoked through stimulation (20–50 Hz; 1-ms biphasic; 1.2× reflex threshold) delivered to the ipsilateral common peroneal nerve through the cuff electrode using a Grass S88 stimulator and isolation unit.

#### State of the Spinal Cord

The motor unit response to tendon vibration was assessed under three different states of the spinal cord: high levels of neuromodulation, acute spinal lesion, and chronic spinal lesion. To increase the activity of lumbar spinal neurons, during one experiment, 50 μL of 100 mM Methoxamine, a norepinephrine α1 agonist, was applied to the spinal cord through the intrathecal catheter. Methoxamine has been shown previously to increase the excitability of spinal motoneurons through an increased magnitude of persistent inward currents (Lee and Heckman, 1999).

In one experiment, a laminectomy was performed from T12 to L1 during the initial surgery. This was packed with saline-soaked gauze, and the incision was held in opposition. Hours after decerebration, the animal was placed back on isoflurane (0.5%), the spine was re-exposed, the dura was cut, and a dorsal hemisection was provided by lesioning the dorsal portion of the cord to the central canal bilaterally. The isoflurane was removed, and the animal was allowed 30 mins to recover.

In one animal, a dorsal hemisection was provided at T13 1 month prior to the terminal experiment. Prior to surgery, cats were sedated (butorphanol, 0.4 mg/kg im; acepromazine, 0.05–0.1 mg/kg im; glycopyrrolate, 0.01 mg/kg sc) and induction was done with propofol (2–3 mg/kg iv). The animal was intubated, and anesthesia was maintained by adjusting isoflurane concentration as needed (1.5–3%). The fur overlying the back and forelimb was shaved with electric clippers. An intravenous line was placed in a cephalic vein to deliver intravenously saline with 2.5% dextrose at a rate of 5–10 ml/kg/h. Under aseptic conditions, a small lower thoracic laminectomy was performed, the dura was removed, and a dorsal hemisection was provided by lesioning the dorsal portion of the cord to the central canal bilaterally. Hemostatic material (Surgicel) was inserted within the gap, and muscles and skin were sutured in anatomic layers. A transdermal fentanyl patch (25 μg/h) was taped to the base of the tail. During surgery and ∼6 h later, an analgesic (buprenorphine, 0.01 mg/kg) was administered subcutaneously. An oral antibiotic (Baytril, 5 mg/kg) was given once a day for 5 days after surgery. The animals were monitored daily by experienced personnel and veterinarians and included manual bladder expressions 1–2 times per day and warm soapy baths as needed.

The TVR data from the cat were compared to a normative dataset of soleus motor unit discharge in the cat (Thompson et al., 2019). This dataset consisted of 297 bouts of self-sustained discharge across 20 animals. These EMG and force data were collected in a similar manner and periods of self-sustained discharge were defined as motor output remaining more than 5 s following the cessation of a specific input.

### Human

#### Ethical Approval

The human data presented here was collected from six adults recruited from the university population. Subjects signed an informed consent approved by the Institutional Review Board of Temple University (protocol #23971).

#### Electromyography and Torque Recordings

Subjects are seated comfortably in an isokinetic dynamometer (Biodex System 4; Shirley, NY, United States) with hips at 45–90 degrees of flexion, the right knee fully extended, and the ankle between 0 and 30 degrees of plantarflexion. The ankle was securely attached to a footplate and which was coupled to a six-degree of freedom load cell (JR3 75E20; Woodland, CA, United States) with the axis of rotation aligned to the center of the ankle joint. EMG signals are collected from the Tibialis Anterior (TA), Soleus (Sol), Medial Gastrocnemious (MG), or Lateral Gastrocnemious (LG) using 64 channel electrodes grids placed on the belly of the muscle.

The output of a single 8.9 mm differential channel (channel 27) of the array was collected through a secondary data acquisition system. Feedback of the rectified and smoothed (500 ms RMS) EMG signal for TA or Sol was provided to the subjects during dorsiflexion or plantarflexion, respectively, on a 42-inch monitor placed ∼1.5 m in front of the subject at eye level.

#### Vibration

A commercially available personal massager (Lyork, Guangdong Sheng, China) was used to evoke TVR in humans by manually holding the vibrator perpendicular to the TA or triceps surae tendon. This consisted of a 3 cm silicone contact with the skin, vibrating at 78 Hz. Vibration frequency was clearly visible in the off-axis force component of the loadcell and used for this calculation. To both promote the TVR response and provide access to the tendon, the tested muscle was slightly lengthened by rotating the ankle joint – to assess the TVR of the TA, the ankle was put into 30 degrees of plantarflexion, whereas the ankle was held neutral when assessing plantarflexion.

#### Experimental Design

Our primary goal with the human data was to quantify the discharge of the same motor unit driven by vibration and by voluntary drive. To accomplish this, we evoked a relatively long TVR response over ∼40 s of vibration, the subject was then provided ∼20 s of rest, afterward the subjects were asked to either dorsiflex or plantarflex in an attempt to volitionally match the conditioned TA or Sol EMG feedback signal from the vibration period. Prior to each trial, the subjects were asked to relax during the vibration and not intervene; following each trial, subjects confirmed that they were relaxed during the vibration.

### Analysis

In both the cat and human, the high-density EMG data was decomposed into the discharge of individual motor units through a well-validated algorithm (Negro et al., 2016). Only units with a silhouette value greater than 0.90 were isolated for further analysis. In the cat, these motor unit discharge times have been demonstrated to have a rate of agreement of 93% among the same motor unit decomposed from both the array and fine wire signal (Thompson et al., 2018).

To quantify the motor unit discharge response to tendon vibration, all the ISIs from all of the motor units are collapsed to form a composite interspike interval (cISI) histogram for each period of interest. This cISI histogram was normalized by dividing each bin by the total number to provide a percentage used for further analysis. For the cat data with a single input and all the human data, a 10–40 s segment of steady state activity was chosen for analysis; for the cat data with multiple inputs, segments of time with a single or combined inputs were calculated. Histogram parameters are relatively wide 20–800 ms range with 2-ms bin width allowing for standardization across trials.

In the cat, the punctuation of the cISI histogram was calculated in two manners. First, a within histogram metric of punctuation was defined as the absolute sum of the first derivative of the histogram (ASD). To calculate the ASD, sequential bin heights were subtracted from one another using the normalized cISI histogram, the absolute value was found and summated. Using this measure, a cISI distribution whose sequential values are relatively close in number, the ASD will be relatively small. In contrast, a cISI histogram that has a large bin-to-bin variance (as observed with the punctuated histograms) will have an increasingly large number. Second, the TVR data from the cat was compared to a normative data set of motor unit discharge patterns collected from the soleus during self-sustained motor unit discharge of the *in vivo* cat. Each of the current cISI histograms was compared to each of the 297 cISI histograms derived from 5,618 spike trains from control trials. To quantify the pairwise similarity between these histograms, each histogram was normalized and mean subtracted. The Euclidian distance between these two normalized, mean-subtracted histograms was calculated to provide a normalized measure of similarity from zero (identical) to one (dissimilar). The TVR trials were grouped as either TVR alone or TVR contaminated with secondary inputs or altered spinal circuitry. The ASD and distance measures were compared to the self-sustained discharge values using a one-way analysis of variance. Significant pairwise differences were assessed using Tukey’s Honest Significant Difference *post hoc* test.

The human data allowed for a similar within and across trial analysis of the discharge characteristics. Normalized cISI histograms are constructed for each muscle within a trial. The ASD is calculated for both the vibration and volitional evoked contractions. Further, within each trial, the vibration and volitional induced motor output is compared by Euclidian distance between the normalized, mean subtracted vibratory and volitional histograms pairs. As we were able to get paired vibration and volitional contractions in the humans, average torque, EMG, and motor unit discharge rate were calculated during the last 5 s of each contraction; in addition, the recruitment threshold was calculated for each motor unit in each contraction. Paired t-tests were performed to assess for differences in torque and motor unit characteristics between each set of vibration and volitional contractions.

## Results

### Cat

To assess the robustness of punctuated discharge of motor units in response to tendon vibration in the cat, we altered the vibration parameters, combined TVR with additional reflexive inputs and assessed TVR with spinal circuits in multiple states.

#### Tendon Vibration Reflex Parameters

**FIGURE 1:**
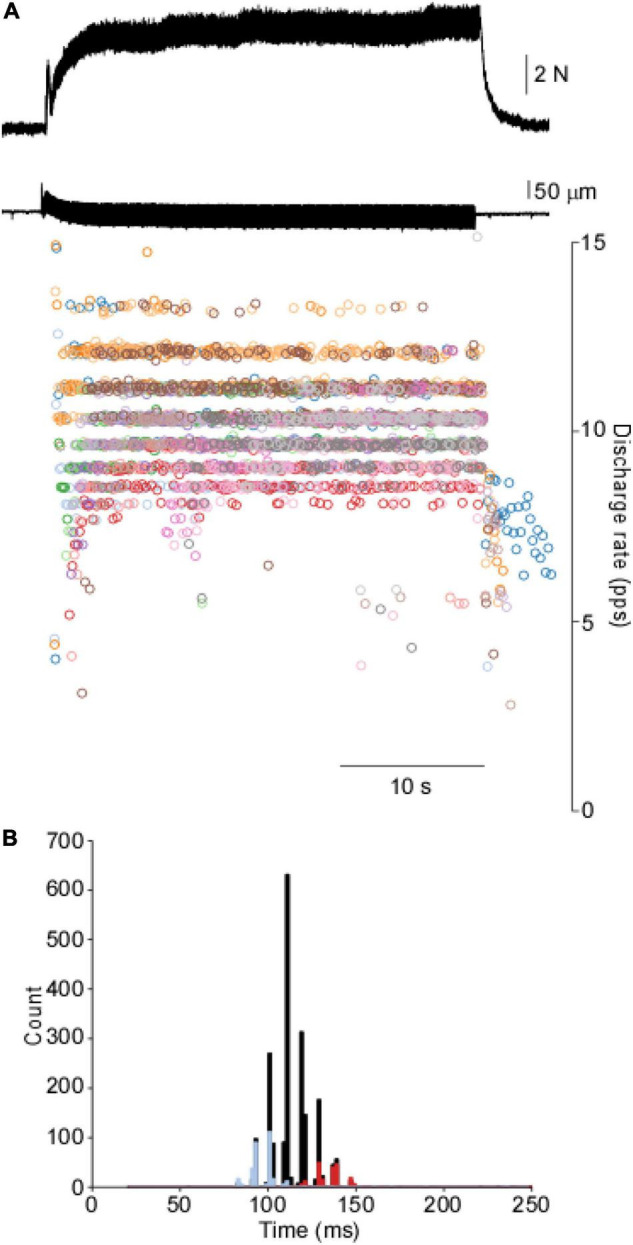
Motor unit response to vibration in the cat’s soleus. **(A)** Tendon vibration of the soleus muscle produces motor output through the punctuated discharge of motor units. **(B)**. The cISI histogram reveals a highly punctuated discharge across a range of intervals. Two motor units are overlaid on top of the cISI histogram to demonstrate a single unit is relatively constrained further from the population data.

Figure 1 shows a representative force and motor unit response to 40 s of tendon vibration. A gradual buildup of muscle force occurs via the recruitment of additional motor units, whereas minimal changes in discharge rate are observed following an initial acceleration of discharge at the onset. When the instantaneous discharge rates for each motor unit are overlaid onto one another, a clear banding of discharge rates is observed. This is further shown in the composite ISI histogram, where abrupt punctuations are observed. ISI histograms of two of the 18 motor units collected during this contraction are plotted on top of the cISI histogram and show individual motor units can be non-overlapping within the range of the cISI histogram.

**FIGURE 2:**
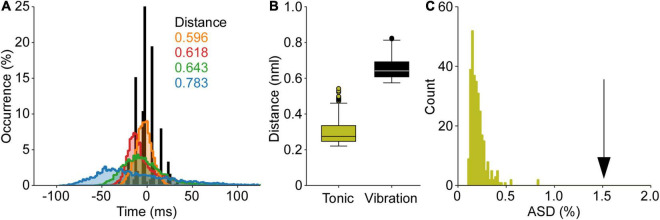
Quantification of punctuated discharge in response to tendon vibration. The data from Figure 1 is used to demonstrate both analyses of punctuated discharge. **(A)** First, the mean subtracted, normalized cISI histogram is compared to a normative dataset of self-sustained discharge. The Euclidian distance is calculated between these histograms. **(B)** The distance between the TVR evoked cISI histogram is greater than the distance between cISI histograms derived from the self-sustained discharge. **(C)** Second, the absolute sum of the first derivative of the cISI histogram (ASD) during vibration is much greater than the ASD values found during self-sustained discharge. Both the distance and ASD measures suggest the TVR cISI histogram is more punctuated than the cISI histogram derived from a control dataset of self-sustained motor unit discharge.

The punctuation of the ISI histogram is quantified in two ways. First, we take the summation of the absolute sum of the sequential difference of the cISI histogram (ASD). The data in Figure 2 demonstrates an ASD value of 1.54. The ASD value observed for this TVR trial is well outside of the range of ASD values derived from the control data (0.21 ± 0.09; mean ± SD). Second, we can compare the TVR evoked histogram to a normative dataset using the Euclidean distance between the normalized histograms. Figure 2 demonstrates the distance from the vibration data to each of the control trials. The control trials demonstrate an average 0.31 ± 0.11 distance from one another, whereas this trial has a mean distance of 0.66 ± 0.06. Both the ASD and the distance measures demonstrate that motor unit discharge patterns in response to tendon vibration in the cat are highly punctuated. This punctuated activity is not observed in the control data, where is an absence of stimulus-evoked motor unit activity.

**FIGURE 3:**
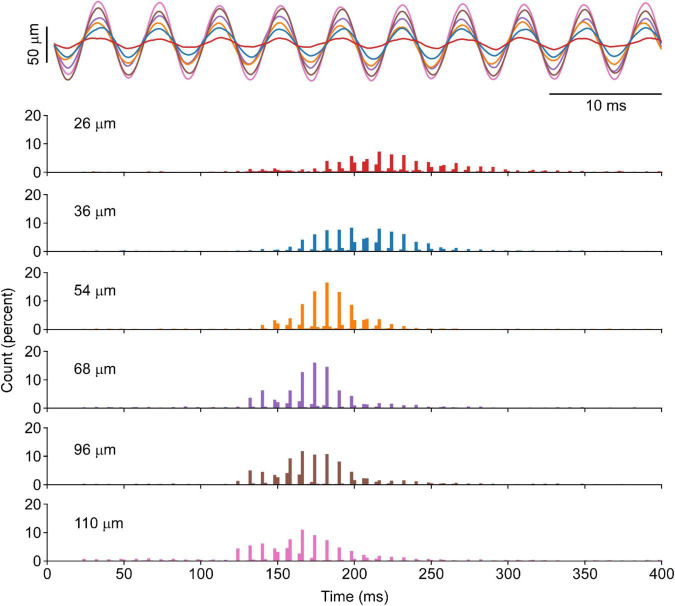
Motor unit response to vibration of different amplitudes in the cat’s soleus. A segment of position recording of vibration excursion overlapped across a range of amplitudes is shown for six different amplitudes at the same vibration frequency in one animal. The corresponding cISI of evoked at each length shown increased vibration amplitude will produce a leftward shift in the ISI, whereas the punctuation remains evident at all vibration amplitudes.

In one animal, the amplitude of the vibration was altered over a ∼100 μm range to quantify the occurrence of the punctuated discharge. Figure 3 provides an example of motor unit response to changes in vibration amplitude. Across the 136 motor unit spike trains, punctuated discharge occurred under each of these amplitudes, with an ASD of 1.56 ± 0.14 and a distance of 0.59 ± 0.05. There was, however, a leftward shift in the ISI histogram with increasing vibration amplitudes.

To better understand the potential effect of the vibration stimulus itself and to better replicate approaches used in humans, a less precise vibrator (electric toothbrush), was manually applied perpendicular to the distal tendon. Such apparatus and setup are more akin to human approaches, as compared to precisely controlled vibration delivered parallel to the muscle typically performed in the cat. Such vibration stimuli produced results qualitatively and quantitatively similar to those evoked by more traditional approaches used elsewhere. The response of 61 motor unit spike trains evoked in response to the manual application of the toothbrush perpendicular to the tendon contains an ASD of 1.18 ± 0.41 and a distance of 0.58 ± 0.04.

#### Tendon Vibration Reflex in Combination With Secondary Synaptic Drive

Next, we added other excitatory and inhibitory inputs in combination with tendon vibration in order to assess if such secondary inputs could serve to diminish the punctuated discharge observed with vibration alone. Electrical stimulation of select peripheral nerves or stretch of the agonist muscle were used to deliver three forms of secondary inputs.

**FIGURE 4:**
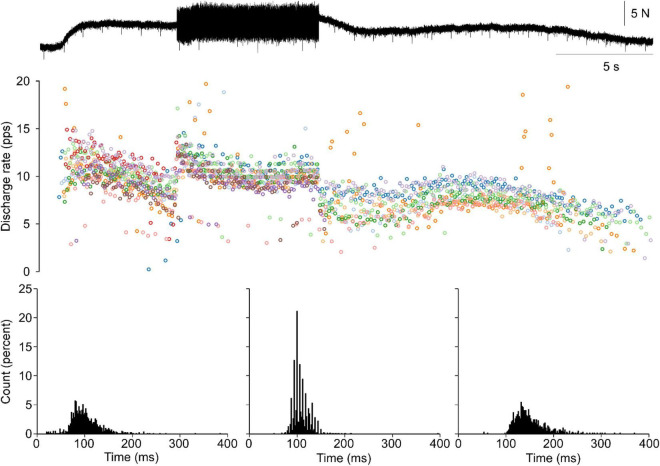
Punctuated discharge remains with the addition of crossed extension. A single trial of relatively brief train of vibration is applied during a longer bout of crossed extension (XEX) stimulation. During XEX alone, no punctuation is observed. Concurrent vibration elicits an increase in both soleus torque and motor unit discharge rates. Furthermore, the discharge during TVR + XEX shows robust punctuation. This is observed in the representative data and the cISI histograms for the XEX alone (sides) and TVR + XEX (middle).

Electrical stimulation of a distal branch of the contralateral superficial peroneal nerve was used to elicit a net excitatory stimulus to the soleus motor pool (XEX; Figure 4). During a prolonged bout of 20 Hz XEX stimulation, 5–10 s long bouts of tendon vibration are delivered. From 68 motor unit spike trains, the ISI histograms derived from the XEX alone portions showed no apparent punctuation in discharge with an ASD of 0.36 ± 0.07 and a distance of 0.30 ± 0.04. However, in the XEX + TVR periods, significant increases in punctuation were observed (ASD = 1.18 ± 0.19 and distance = 0.47 ± 0.08). Punctuation is observed in the XEX + TVR periods but not in the XEX alone epochs.

Next, during the vibration period, a 2 mm peak-to-peak change in muscle length is delivered at 1 Hz. Motor units are responsive to stretch, as demonstrated by their change in discharge with muscle length. However, very little difference is observed in the ASD (1.23 ± 0.06 and 1.21 ± 0.08) and distance (0.52 ± 0.04 and 0.58 ± 0.09) values between the TVR alone and Stretch + TVR conditions from 122 motor unit spike trains, with punctuation being demonstrated throughout.

Lastly, electrical stimulation of the nerve to TA was used to provide reciprocal inhibitory synaptic input to the soleus motor pool. As with the stretch condition, a long bout of TVR is interposed by ∼10 s train of electrical stimulation of the common peroneal nerve. This was observed to decrease ongoing force and EMG activity and was mediated by either motor unit derecruitment or a decreased discharge frequency of ongoing motor units. Therefore, during the inhibition + TVR periods, a decreased frequency of discharge is observed. However, data from 63 motor unit spike trains, there is little change between the punctuation observed in the TVR and inhibition + TVR conditions with ASD values of and 1.51 ± 0.07 and 1.50 ± 0.10 and distance values of 0.53 ± 0.05 and 0.67 ± 0.06, respectively.

#### Tendon Vibration Reflex in Altered States of the Spinal Cord

In our final attempt to quantify the robustness of motor unit response to TVR in the cat, we sought to quantify the TVR evoked motor unit responses to altered states of spinal circuitry through pharmacological and surgical approaches. First, exogenous neuromodulation of spinal neurons can profoundly alter the state of spinal circuitry. The NEa1 receptor agonist methoxamine has been shown to increase the excitability of spinal motoneurons (Lee and Heckman, 1996). In this state, the increased intrinsic currents and receptiveness to other inputs may serve to diminish the proportional role of the vibratory input. Despite this, motor units can respond to vibration, and this response maintains its punctuated discharge. From the 33 motor unit spike trains collected in this condition showed ASD and distance values of 1.08 ± 0.11 and 0.49 ± 0.09, respectively.

Additionally, spinal lesions in both the acute and chronic stages will have a profound impact on the state of spinal circuitry. Though motor unit discharge rates and force were substantially lower, an acute dorsal hemisection had little effect on the punctuated discharge (ASD = 1.35 ± 0.07 and distance = 0.75 ± 0.18) across 82 motor unit spike trains. In an animal with a chronic dorsal hemisection, punctuation remained strong (ASD = 0.96 ± 0.28 and distance = 0.57 ± 0.10) across 53 motor unit spike trains.

#### Overall Comparison in the Cat

In the cat, every application of vibration to the soleus tendon resulted in motor output that was highly punctuated. Such punctuation was never observed during self-sustained discharge. Across all 24 epochs of TVR alone from four animals, the average ASD was 1.44 ± 0.15 and the average distance was 0.56 ± 0.05. Across the 18 epochs of TVR contaminated with either secondary inputs or altered spinal circuitry from six animals, the average ASD was slightly smaller (1.21 ± 0.23) and the average distance was slightly larger (0.59 ± 0.14). When the TVR alone and contaminated TVR groups were compared to the 297 self-sustained discharge trials, both the ASD and distance measure revealed a similar trend. Separate one-way ANOVAs revealed self-sustained discharge has a significantly lower ASD and distance compared to both TVR alone and the corrupted TVR (all *p* < 0.0001). Significant differences were observed between TVR alone and the corrupted TVR for ASD (*p* < 0.0001) but not distance (*p* = 0.77). Though significant, the difference between the TVR alone and contaminated TVR on the ASD measure was much smaller (0.23) than the difference between these data and the self-sustained discharge (1.23 and 1.00, respectively).

### Human

Six individuals (two female) with an average age of 22.8 ± 5.2 years, height of 171.6 ± 10.3 cm, and weight of 68.9 ± 10.9 kg participated in the experiment. The human data was collected and analyzed in a manner akin to both previous human investigations and the above cat experiments, however, the human data provided us with the ability to have the participants volitionally match the EMG evoked during the prior tendon vibration.

The mean EMG amplitude was not different between vibration and volitional contractions (26.2 ± 40.3 versus 25.8 ± 40.3 μV; *p* = 0.822). However, this matched EMG resulted in greater ankle torque in the voluntary (5.27 ± 6.84 Nm) as compared to the vibration (3.78 ± 5.86) contractions (*p* = 0.012).

From the surface EMG arrays, 67 motor unit spike trains were matched across both the vibration and volitional conditions. This represented 44 TA motor units across 10 dorsiflexion trials from four individuals and 8 Sol, 6 LG, and 9 MG motor units across 8 trials in two individuals. On average, across all motor units the discharge rates (9.5 ± 2.7 versus 9.5 ± 3.2 pps) and recruitment thresholds (2.22 ± 3.99 vs. 2.01 ± 2.29 Nm) were not different between vibration and volitional activation (*p* = 0.953 and *p* = 0.586, respectively).

**FIGURE 5:**
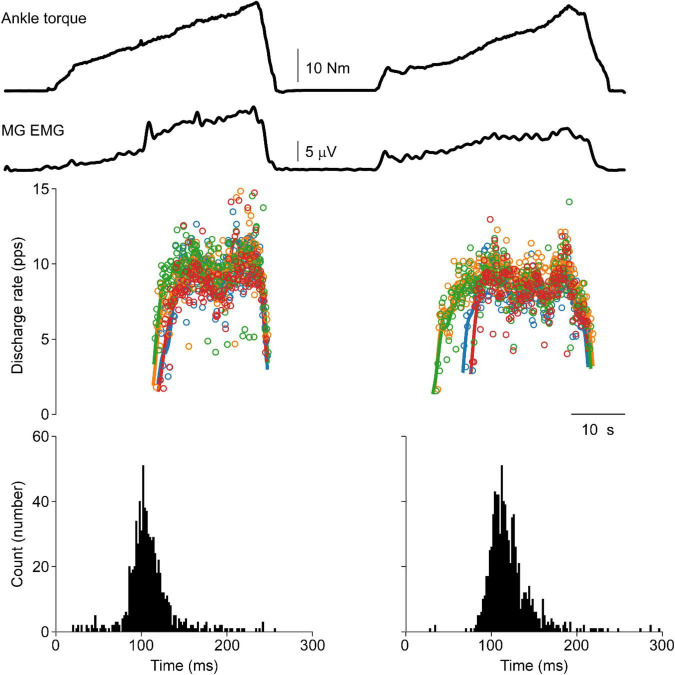
Vibration evoked and matched volitional contractions in the human. Representative data from a single human participant. The first contraction is elicited by vibration of the triceps surae tendon, whereas the second contraction is a volitional contraction set to match the EMG profile of the vibration evoked contraction. Similar motor unit discharge patterns are observed in both contractions. No punctuation is observed during the vibration evoked contraction with ASD values of 0.36 and 0.32 during vibration-evoked and volitional contractions, respectively. A distance of 0.28 is observed between these cISI histograms.

Moreover, punctuated motor unit discharges evoked through tendon vibration were not observed in the human lower limb. Figure 5 shows ankle torque, MG EMG, and four MG motor units during vibration evoked contraction of the triceps surae followed by a volitional match of the EMG produced during the vibration period. A lack of punctuated discharge is observed in the TVR evoked motor unit discharge.

This lack of punctuated discharge is consistently observed in the lower limb of subjects. Data from these six subjects consistently demonstrates this lack of punctuated discharge in response to focal TVR at the ankle. ASD values were nearly identical between the vibration (0.25 ± 0.08) and volitional (0.25 ± 0.06) histograms (*p* = 0.93). The average distance between the vibration and volitional contraction is 0.27 ± 0.14, slightly below the distance between the self-sustained discharge condition in the cat.

**FIGURE 6:**
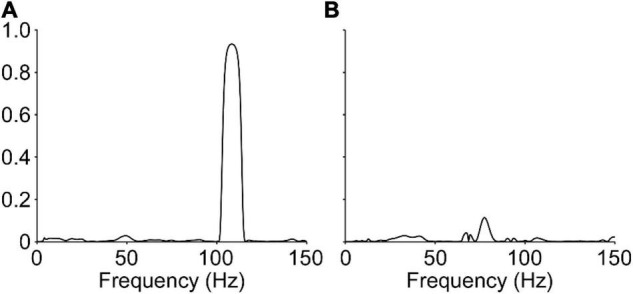
Coherence between the neural drive to muscle and the vibration frequency. Representative data showing the coherence between the composite spike train and the vibration frequency, determined through the force signal, are shown for the cat **(A)** and human **(B)**. The data from the cat show relatively large coherence (0.93) at the vibration frequency, however, data from the human shows a stark reduction, but not complete abolishment, of the observed coherence (0.11) at the vibration frequency.

Despite this lack of punctuated discharge, the neural representation of the vibration frequency was not fully absent in humans. Figure 6 shows the coherence between the CST and the unfiltered force or torque output for one representative example of the cat and human data. As expected, the CST extracted from all cat recordings is highly coherent with the vibration frequency (>0.8). On the other hand, human data show a much lower amplitude, though not absent, coherence at the vibration frequency (<0.2).

## Discussion

The punctuated motor unit discharge in response to tendon vibration is a robust occurrence in the cat. However, in humans, such punctuated discharge is not readily apparent.

### Tendon Vibration in the Cat

The TVR has been thoroughly investigated in the cat. Spindle afferents are highly sensitive to vibration and result in afferent action potentials time locked to the vibration period (Brown et al., 1967). This afferent drive provides distributed monosynaptic input to each of the motoneurons of the motor pool (Mendell and Henneman, 1968) and steady-state activation of the Ia afferents will produce a relatively stable depolarization of the spinal motoneuron (Lee and Heckman, 1996). Previous data has shown spinal motoneurons in the cat discharge at integer multiples of the vibration frequency. Here we demonstrate this is a common occurrence for the soleus muscle across a wide range of conditions.

It is possible that our vibration was not fully selective for Ia afferents. Group II afferents may be activated by tendon vibration in the cat, however, it is expected that the discharge of these afferents would be proportional to the amplitude, rather than frequency, of vibration (Brown et al., 1967). As the muscle was surgically isolated from the surrounding tissue, it is likely that potential heteronymous and cutaneous activation is diminished but cannot be excluded. Given the strict rule-like nature of the motoneuron discharge, it may be expected that recurrent inhibition would be patterned in a similar manner. Lastly, Ib fibers may be activated by the stimulus, but would also be activated in a manner that is proportional to the active muscle force generation. While it is clear that the activation of these other pathways does not fully interfere with the punctuated discharge of motoneurons, it remains unclear what role they may have in modulating, or even promoting, this pattern of discharge.

To further explore this idea, we augmented non-Ia sources of synaptic drive to these spinal motoneurons by providing specific afferent drive in conjunction with the tendon vibration. The addition of stretch, crossed extension, and reciprocal inhibitory inputs had clear effects on the mean discharge frequency, however, minimal effects were observed on the punctuated discharges. Similarly, concurrent electrical activation of the nerve to TA (the antagonist muscle) did not alter the discharge pattern in response to tendon vibration of the soleus muscle. Lastly, both pharmacological and surgical attempts to alter the state of the spinal cord failed to significantly diminish the presence of punctuated TVR evoked discharge in the cat.

### Tendon Vibration in the Human

Focal tendon vibration failed to produce punctuated discharge patterns in human lower leg motor units. When quantifying the discharge of the same motor unit across conditions, the TVR evoked contraction, and the volitional contraction produced nearly identical patterns of motor output. This was consistently observed in all units detected in the lower limb in each of the human subjects assessed.

Though researchers have observed some level of phase-locking (Homma et al., 1971; Desmedt and Godaux, 1975; Burke and Schiller, 1976; Hagbarth et al., 1976; Romaiguere et al., 1991) of, numerous other descriptions of human motor unit discharge during focal tendon vibration either did not observe this discharge pattern or did not report it. In the latter group, it may be expected that these investigators would have noted such patterns, as it is quite striking when observing the discharge rate overtime or the ISI histogram. Additionally, a visual assessment of the discharge patterns in these manuscripts does not demonstrate punctuated discharge (Bongiovanni et al., 1990; Bongiovanni and Hagbarth, 1990; Romaiguere et al., 1993; Kiehn and Eken, 1997; Gorassini et al., 2002, 2004; Grande and Cafarelli, 2003; MacDonell et al., 2010; Fuglevand et al., 2015; Mosier et al., 2017). The primary finding from the human data is that the punctuation of motor unit discharge is much less robust than in the cat.

### Potential Mechanisms

Several potential mechanisms may underlie this discrepancy in motor unit discharge patterns in leg muscles in both the cat and human. The discharge of spindle afferents is phase locked to the primary or subharmonic frequency of vibration in both the cat and humans. At sub motor threshold levels, human spinal afferents may not discharge in a non-time locked manner (Fallon and Macefield, 2007), however, at higher vibration amplitudes, this discharge is locked to the vibratory stimulus (Burke et al., 1976; Houk, 1976). If such afferent discharge is phase locked to the vibratory input and if the one-to-all distribution of Ia afferents to spinal motoneuron also holds true, the discrepancy between the cat and human may be due to either activation of non-monosynaptic pathways or length of the reflex arc.

The gradual build-up of force generation during constant vibratory input was initially taken as evidence of the slow activation of polysynaptic circuits through collaterals from the Ia afferents, which are thought to be “more insecure and less straightforward in nature than the monosynaptic excitation” (Godaux and Desmedt, 1975). Though such collaterals exist (Scheibel and Scheibel, 1969; Brown and Fyffe, 1978; Ishizuka et al., 1979; Vincent et al., 2017; Lucas-Osma et al., 2018) and activation of the motoneuron through Ia mediated polysynaptic circuits likely does occur (Jankowska et al., 1981; Burke et al., 1984), the role of the persistent inward currents, intrinsic to the motoneuron, seems highly plausible. Rather than the gradual buildup of polysynaptic circuit activity, the gradual warm-up of PIC activity may underlie this recruitment of additional motoneurons (Heckman and Lee, 2001). In addition to promoting a recruitment-based strategy for increasing torque generation, this secondary form of input, whether extrinsic or intrinsic, may diminish the punctuated discharge of motor units. The motor unit data from the cat presented here demonstrate that secondary forms of input do little to alter the punctuated discharge of spinal motoneurons. Instead, the TVR evoked punctuated discharge remains robust across either patterned afferent drive or alterations in the state of the spinal cord.

Small changes in the dispersion of axonal conduction velocity can affect the transmission of oscillations over longer pathways. If the range of the latency jitter delays of Ia afferents exceeds the vibration period, the motoneurons may receive a smoothed and relatively more uniform synaptic input. Such phenomenon is consistent with the substantial decrease of the correlation between neural drive and vibration oscillations in humans. In humans, the TVR evoked synchronization of motor units may be less readily observed in more distal muscles (Hagbarth et al., 1976). Such findings are consistent with both the cat and human data presented here. However, this is contrary to the patterns of motor unit synchronization during volitional contractions. During voluntary contractions, more distal motor pools are observed to have a greater amount of synchronization (Keen et al., 2012) and shared oscillations in alpha and beta frequency bands (Negro and Farina, 2012; Castronovo et al., 2015). Therefore, it is unclear if the same results will be observed during a stochastic/non-periodic, lower frequency physiological activation of spinal motoneurons.

There are several limitations to these results. First, the cat data were obtained from a single muscle following decerebration. An ideal model would be test this in a variety of muscles in the awake behaving cat, like what was reported here for the human lower limb. Future investigations should work toward developing technical and behavioral approaches to investigate motor unit activity during tonic sensory drive in the awake behaving animal. Additionally, vibration was delivered at different frequencies, however, previous work from the cat has shown that motor unit discharge remains highly punctuated across a range of frequencies (Thompson et al., 2018) and it does not appear that there is a dramatic shift toward punctuated discharge across a range of vibration frequencies in human lower limb muscles (Mildren et al., 2019). Lastly, we may be recording from a biased sample of motor units, particularly in the human. The high-density EMG approach is selective to superficial motor unit and it may be that larger motor units are positioned more superficially (Knight and Kamen, 2005). It would be of great interest to see if there is a subpopulation of motor units which does demonstrate punctuated discharge in response to tendon vibration, however, none of the 67 motor unit spike trains collected from the human lower limb demonstrated this behavior.

Here we observe that focal tendon vibration in the cat produces highly punctuated motor unit discharge. This occurs across various vibration parameters, with the addition of secondary inputs, and with altered states of the spinal cord. This rule-like response to tendon vibration is observed in every unit of every animal. The addition of non-monosynaptic inputs does little to distort the punctuated discharge of spinal motoneurons in response to tendon vibration in the decerebrate cat. Such punctuated discharge is not observed in the human limb. Taken together, these data suggest the lack of punctuated discharge in humans may be due to phenomena other than non-monosynaptic inputs. It may be the case that the dispersion of the coherence between the neural drive and the vibration oscillations is influenced by the long conductance distance between the vibrated tendon and its motoneuron pool.

## Data Availability Statement

The raw data supporting the conclusions of this article will be made available by the authors, without undue reservation.

## Ethics Statement

The studies involving human participants were reviewed and approved by Temple University’s Human Research Protection Program (HRPP). The patients/participants provided their written informed consent to participate in this study. The animal study was reviewed and approved by Institutional Animal Care and Use Committee (IACUC).

## Author Contributions

CT, MJ, DF, CH, and FN contributed to conception and design of the study. CT and MJ performed the experiments. CT and FN performed the analyses. CT wrote the first draft of the manuscript. All authors contributed to manuscript revision, read, and approved the submitted version.

## Conflict of Interest

The authors declare that the research was conducted in the absence of any commercial or financial relationships that could be construed as a potential conflict of interest.

## Publisher’s Note

All claims expressed in this article are solely those of the authors and do not necessarily represent those of their affiliated organizations, or those of the publisher, the editors and the reviewers. Any product that may be evaluated in this article, or claim that may be made by its manufacturer, is not guaranteed or endorsed by the publisher.
